# ID 188 - Efeitos do Uso de Palmilhas nas Funções da Marcha, Equilíbrio e Controle Postural de Indivíduos Pós-Acidente Vascular Cerebral: uma revisão sistemática com metanálise

**DOI:** 10.5327/2237-9622.2025.v34s1.188

**Published:** 2025-11-25

**Authors:** Ketinlly Yasmyne Nascimento Martins, Bárbara Sousa dos Santos, Ana Cheile de Melo Costa, Ana Carolina Rodrigues Alves, Anna Kellssya Leite Filgueira, Kátia Elizabete Galdino

**Keywords:** acidente vascular cerebral, equipamentos ortopédicos, equilíbrio postural, análise da marcha

## Abstract

**Introdução:**

Entre as consequências de um acidente vascular cerebral (AVC), destaca-se o comprometimento do sistema somatossensorial do complexo tornozelo-pé, que afeta negativamente o equilíbrio, o controle postural e a marcha. A manipulação de superfícies sob os pés, por meio de intervenções como palmilhas (órteses plantares), tem sido estudada nessa população para melhorar a qualidade da informação sensorial, através da distribuição da descarga de peso, correção de alterações biomecânicas e estimulação proprioceptiva. No entanto, os benefícios dessas órteses ainda são incertos. Diante disso, o objetivo desta revisão foi investigar os efeitos do uso de palmilhas nas funções da marcha, equilíbrio e controle postural de indivíduos pós-AVC.

**Método:**

Estudo do tipo Revisão Sistemática com Metanálise (PROSPERO ID = CRD42022352629). A pergunta norteadora da pesquisa foi estruturada pelo acrônimo PICOT e incluiu Ensaios Clínicos Randomizados (ECRs) com pacientes adultos pós-AVC, capazes de caminhar com ou sem assistência. A intervenção consistiu no uso de palmilhas projetadas para população, comparadas a palmilhas sham ou intervenção de base, nas quais se avaliou a eficácia da tecnologia na melhoria da marcha, equilíbrio e controle postural. As fontes de evidências foram as bases de dados LILACS, CENTRAL, PubMed, Embase, Scopus e PEDro, além de registros de ECRs e Literatura Cinzenta.

**Resultados:**

A revisão foi composta por nove ECRs (sendo dois incluídos na metanálise), constituindo uma amostra de 265 participantes, dos quais 68% (n=180) encontravam-se na fase crônica. Foram identificados quatro tipos de palmilhas: postural, texturizada, de elevação, e com feedback auditivo. Apenas um estudo padronizou os calçados dos participantes. Os achados indicaram uma pequena ou nenhuma diferença entre os grupos, exceto com a palmilha de feedback auditivo, que apresentou o melhor efeito na melhoria do equilíbrio, aumentando o desempenho no Berg Balance Scale (BBS). A metanálise demonstrou que o uso da palmilha com feedback auditivo aumentou em 6.07 cm/s a média da velocidade da marcha (p=0.07). A tecnologia também aumentou a pontuação do BBS em 3.22 pontos (p=0.04). Da mesma forma, a intervenção diminuiu o tempo de execução do Timed up and Go (TuG) em 3.53s (p=0.02). A certeza da evidência foi de muito baixa à moderada.

**Conclusão:**

Os resultados da metanálise apontaram que o aumento da velocidade da marcha não foi estatisticamente significativo. No entanto, a intervenção diminuiu o tempo de execução do TuG, o suficiente para promover mudanças na mobilidade (mínima mudança detectável = 2.9s). Contudo, o tamanho de efeito alcançado não foi suficiente para atingir valores abaixo do ponto de corte para reduzir o risco de quedas (n <14s). Em relação ao BBS, o aumento da pontuação não foi clinicamente significativo, pois são necessários seis pontos de variação para ter 90% de confiança de uma mudança genuína no equilíbrio. Na síntese qualitativa observou-se em sua maioria pequena ou nenhuma diferença entre os grupos, com tamanho do efeito variando entre os diferentes tipos de palmilhas e subgrupos de pacientes. Entre as limitações encontram-se a heterogeneidade dos estudos, a falta de padronização dos calçados e a impossibilidade de correlacionar as fases do AVC com os resultados. Assim, a evidência atual sugere que, embora promissoras, as palmilhas ainda não apresentam impacto clínico significativo para melhorar os desfechos investigados.

